# Some Errors about Cancer of the Breast

**Published:** 1900-04-07

**Authors:** 


### SOME ERRORS ABOUT CANCER OF THE BREAST.

Sir William Banks, in his Lettsomian Lectures, points out five errors which he speaks of as popular errors in regard to cancer to which the public and, he regrets to say, not a few medical men still give credit. They are partly the result of statements made in text-books and not properly explained, and partly the result of popular tradition. First about heredity. The idea that cancer is hereditary is dangerous, in that it leads a woman with a growth in her breast, in the absence of any cancerous taint among her relatives, to comfort herself with the idea that it cannot be cancer as such a thing was never known in her family, and thus leads to delay. Second, about pain. This error is a most serious one. The termination of all external cancers is invariably accompanied by severe suffering, and these dreadful cases appeal so distressingly to the minds of women that they always associate pain and cancer. But, unfortunately, there are no neoplasms which produce less pain in their early stages than cancer, and thus, since to the female mind the absence of pain means the absence of cancer, we have delay again. So far as the early stage is concerned it may indeed be laid down that the presence of pain goes against a recent lump being cancerous. Third, about cachexia. This is an error which is chiefly made by doctors. Often, says Sir William Banks, he has been struck by a medical friend remarking in regard to some patient, "Well, but look at her ruddy colour and healthy aspect. There is no cancerous cachexia there." Like pain, we have here an old superstition, a deeply-rooted popular belief to the effect that a patient who has a cancer must have a pallid, emaciated, haggard appearance. When fœtid discharge and agonising pain and the other consequences of cancer have done their work, then it is true that the unfortunate patient does present the appearance of a cachexia, but certainly not at the beginning of her disease. The victims of cancer are usually well-nourished persons, with a fine healthy colour in their cheeks. It is to be admitted that in certain forms of intestinal and other internal cancers, where the disease is long latent, the presence of cachexia is an important point; but such are not early cases. Fourth, about nipple retraction. This, again, is an error made by the doctor, and it results from their having been misled by the text books, which have given out too broadly that retraction of the nipple is a sign of carcinoma, while they have not sufficiently qualified the statement by clearly indicating that the sign is only of value when the disease happens to be situated immediately beneath the nipple. Truly, retraction of the nipple is practically pathognomonic when the disease lies beneath it, but its absence means nothing where the disease is situated away from the centre of the gland. Fifth, about adhesion of the skin. This is a grave indication of sublying cancer. It does not occur with any other tumour except an inflammatory swelling of the nature of an abscess. In such cases the skin is fairly smooth over the abscess of inflammatory swelling. But over a carcinoma "if you try to pinch the skin up between the finger and thumb immediately a number of fine points appear, which are the hair follicles caught and tucked down by the cancer." This appearance is almost certainly characteristic of subjacent cancer. This is a matter of importance, since one of the conditions which most closely stimulate mammary cancer is chronic mastitis.

